# Impact of *Coxiella burnetii-associated* abortions in Finnish forest reindeer within a zoo on public health appeared negligible succeeding mitigation strategies

**DOI:** 10.3389/fvets.2026.1882481

**Published:** 2026-08-25

**Authors:** Marleen M. Kannekens-Jager, Andries A. Kampfraath, Christine Kaandorp, Annemieke Dinkla, Frank Harders, Lars Ravesloot, Pieter Jacobs, Judith M. A. van den Brand, Erik A. W. S. Weerts, Senta L. D. Janssen, Lenny M. J. Engelen, Amanja Verhaegh-Haasnoot, Robin Ruuls, Diederik Brandwagt, Marloes Heijne, Remco Dijkman, Arne Vanhoudt, Liesbeth Harkema, Ad P. Koets, René van den Brom

**Affiliations:** 1 Department Bacteriology, Host-Pathogen Interaction and Diagnostic Development, Wageningen Bioveterinary Research, Lelystad, Netherlands; 2 Department of Veterinary Integrative Data Analytics, Wageningen Bioveterinary Research, Lelystad, Netherlands; 3 GaiaZOO, Kerkrade, Netherlands; 4 Diergaarde Blijdorp, Rotterdam, Netherlands; 5 Dutch Food and Consumer Product Safety Authority, Utrecht, Netherlands; 6 Veterinary Pathology Diagnostic Centre, Division of Pathology, Faculty of Veterinary Medicine, Utrecht University, Utrecht, Netherlands; 7 Department of General Healthcare, South Limburg Public Health Service, Heerlen, Netherlands; 8 Department of Social Medicine, Care and Public Health Research Institute (CAPHRI), Maastricht University, Maastricht UMC+, Maastricht, Netherlands; 9 Diagnostic and Crisis Organization, Wageningen Bioveterinary Research, Lelystad, Netherlands; 10 National Institute for Public Health and the Environment (RIVM), Centre for Infectious Disease Control (CIB), Bilthoven, Netherlands; 11 Royal GD, Deventer, Netherlands

**Keywords:** abortion, *Coxiella burnetii*, genotyping, mitigation, one health, reindeer, WGS

## Abstract

*Coxiella burnetii* is a Gram-negative, ubiquitous bacterium that may infect many animal species as well as humans. In this study, transmission of *C. burnetii* during an outbreak of abortions in a herd of Finnish forest reindeer (*Rangifer tarandus fennicus*) in a Dutch zoo was investigated. Diagnostic sampling showed positive *C. burnetii* antigen detection with qPCR from vaginal swabs and positive *C. burnetii* serology (ELISA) in all cases of abortion (4/4) and in one animal after normal parturition. Placental tissue (*n* = 2) and fetal liver tissue (*n* = 1) retrieved after two abortions were positive for *C. burnetii* infection by qPCR and immunohistochemistry. Histological staining revealed the presence of *C. burnetii* in the placenta, indicating infection. Follow-up diagnostic approaches were aimed toward isolation, strain typing, and molecular analysis. Direct sequencing from placental tissue resulted in a full genome and plasmid sequence. Whole-genome sequence analysis, including multilocus variable-number tandem repeat analysis (MLVA), resulted in similarities with a *C. burnetii* strain previously found in cattle. A One Health approach was implemented, guiding efforts to prevent and monitor human Q fever following potential exposure to *C. burnetii*. No human Q fever cases were reported, and additional serological testing of exposed zoo personnel showed no indications of infection. This article describes the first documented case of clinical abortions caused by *C. burnetii* in Finnish forest reindeer, offering new insights into coxiellosis in non-domestic animals and highlighting lessons to inform future preparedness and response.

## 1 Introduction

*Coxiella burnetii* is a Gram-negative, intracellular bacterium that causes coxiellosis in animals and Q fever in humans (1). *C. burnetti* has a nearly global distribution (2). It infects a wide range of hosts, including ticks, birds, and various mammals, and is well known for causing reproductive disorders in domestic ruminants (3). In pregnant goats and sheep, abortion during late gestation is the predominant clinical manifestation, whereas cattle typically show fewer clinical signs, although abortions, subfertility, and metritis may occur (4). In humans, acute Q fever usually presents as a self-limiting febrile illness, atypical pneumonia, or hepatitis. It is estimated that 1–5% of patients may progress to chronic Q fever, most commonly endocarditis, which accounts for 60–70% of chronic cases (5).

The worldwide prevalence of infection in domestic ruminants shows a higher prevalence in cattle (20% at animal level and 38% at herd level) than in small ruminants (around 15% at animal level and 25% at herd level) (6). When pregnant goats are infected, extremely high bacterial loads may be shed during parturition or abortion via placental tissues and fetal fluids (7). This massive shedding during and shortly after parturition is the most important source of environmental contamination and transmission to other animals and humans by inhalation of contaminated aerosols or dusts containing *C. burnetii* (8–10).

The public health relevance of *C. burnetii* was highlighted during the largest Q fever outbreak recorded to date, which occurred in the Netherlands between 2007 and 2010 and resulted in more than 4,000 human cases (5, 11). Epidemiological investigations and molecular analyses using multilocus variable-number tandem repeat analysis (MLVA) identified *C. burnetii* shedding dairy goats as the main source of the outbreak (12). Further molecular analysis identified critical genes associated with increased virulence in the outbreak goat strain, *C. burnetii* strain (NL3262), which may have contributed to the scale of the outbreak (13). As a measure during the outbreak, Q fever became notifiable in small ruminants in the Netherlands in 2008.

In spring 2025, in a zoo in the Netherlands, a group of captive Finnish forest reindeer (*Rangifer tarandus fennicus*) experienced abortions due to *C. burnetii.* The detection in a novel host species in a public site raised concerns from both veterinary and public health authorities, particularly considering the historical Dutch Q fever outbreak. This research report aims to assess the risks associated with this outbreak in captive Finnish forest reindeer, highlight key aspects of diagnostics and molecular investigations, assess mitigation measures to prevent transmission, and assess clinical decision-making within a One Health framework. By integrating knowledge and experience from both veterinary and public health experts, this study seeks to inform future preparedness and response to zoonotic threats in public settings.

## 2 Methods

### 2.1 Animals

#### 2.1.1 Background herd

A zoo-based herd of 10 Finnish forest reindeer, 1 male (M1) and 9 does (D1–D9), was housed in a tundra-simulating enclosure with an elevated boardwalk pathway allowing visitors to traverse the habitat overhead. Finnish forest reindeer are listed as a vulnerable animal species (14).

**Figure 1:**
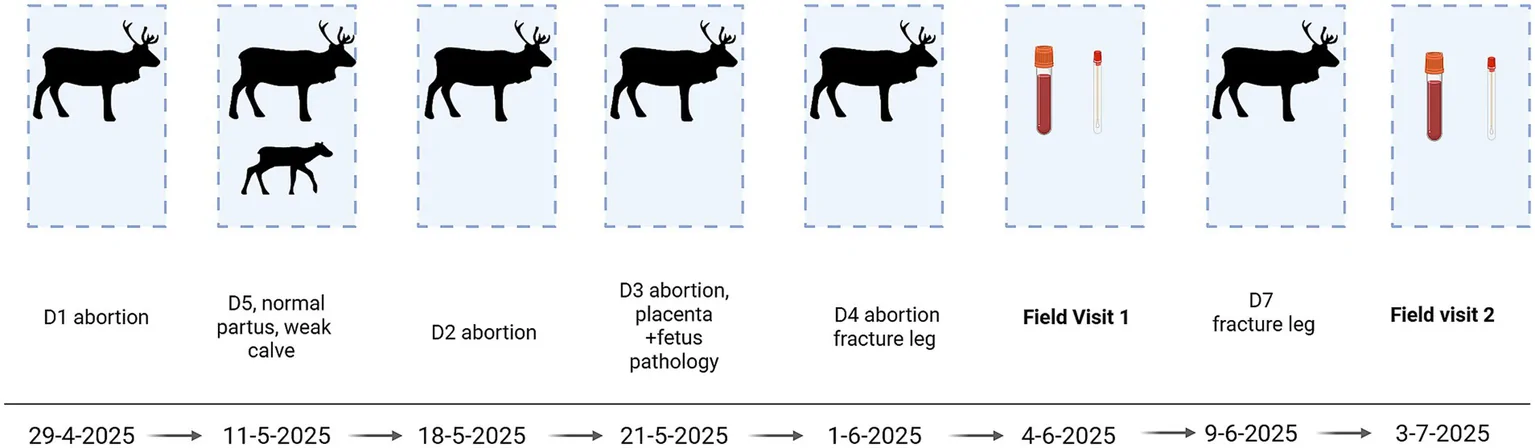
Timeline of events for Finnish forest reindeer in the zoo. Created in BioRender. Kannekens-Jager, M. (2026) https://BioRender.com/ofdxz3o.

From the six pregnant females, four females (D1–D4) aborted within a 5-week period between April and June 2025. One female (D5) had a normal parturition with a healthy but weak calf, and one female (D6) was due to calf. Primarily pathologic examinations were executed by the Veterinary Pathology Diagnostic Centre (VPDC) on one calf and the placenta of D3. The zoo veterinarian additionally submitted blood for serology and vaginal swabs from the reindeer to enable differentiating pathogens (including *Clamydia abortus, Neosporum caninum*, and *Leptospira interrogans*) causing abortions, and this resulted in positive Q fever serology and antigen detection with qPCR for *C. burnetii* at Royal GD. Although Q fever in reindeer was not notifiable at that time, the zoo veterinarian decided to inform the competent authorities. The zoo took immediate preventive measures, including isolation of the animals and restriction of public access because of the zoonotic nature of the pathogen. The animals that aborted were separated from other animals and could not come into contact with visitors. Animal caretakers used personal protective equipment (PPE) and included strict hygiene protocols. After abortion, the placental materials were removed, and the area was cleaned and disinfected with Halamid ®. Two field visits at the zoo were performed for official sampling and further preventive measures, see timeline Figure 1.

#### 2.1.2 Data collection

Veterinarians took official regulatory samples (blood and vaginal swabs) from the animals, and these were submitted to Wageningen Bioveterinary Research (WBVR) for confirmation. Placental membranes and one aborted fetus (D3) were tested for the presence of *C. burnetii*, and placental tissue was used for isolation and molecular identification. The remaining animals were followed in time and tested serologically as well as by PCR (vaginal swab and blood) after 4 weeks. Unfortunately, two animals (D4 and D7) were lost for follow-up; these animals were euthanized after they had fractured a leg due to stress of handling. D7 was submitted for postmortem examination. Animal details are in Supplementary Table S1.

#### 2.1.3 Diagnostic testing of animal samples

##### 2.1.3.1 Polymerase chain reaction (PCR)

Pathogen DNA detection was performed with a real-time PCR (qPCR) test based on the detection of the transposase gene IS1111 according to the method described by Klee et al. (15).

##### 2.1.3.2 Serology

Antibody detection was performed by indirect ELISAs with PrioCHECK Ruminant Q Fever Ab Plate kit® (ThermoFisher Scientific). Serum dilutions were prepared, and Optical density (OD) was measured with spectrophotometry (450 nm). S/P % were calculated; S/P > 40% was considered a positive result and S/P ≤ 40% a negative result as per the manufacturer’s instructions.

##### 2.1.3.3 Pathological examination and immunohistochemical staining

Routine pathological investigation was performed on the calf and placenta (D3) at VPDC. IHC was performed and analysed by GD and VPDC. Necropsy of a young female (D7) was performed at WBVR.

#### 2.1.4 Molecular analysis of *C. burnetii*

##### 2.1.4.1 Multilocus variable-number tandem repeat analysis (MLVA)

MLVA (wet method) was performed on a selection of the described loci to genotype *C. burnetii* isolates by determining the number of tandem repeats at multiple loci described by (35), using extended primer sets described by Arricau-Bouvery et al. (16) and Klaassen et al. (12). MLVA was performed on official samples (*n* = 6) from animals D1–D5 and analyzed together with NineMile reference strain (Nm RSA 493), a cattle strain (cattle GB), and a negative control. MLVA was performed on Multilocus (MS) MS 01, MS 03, MS 21, MS 22, MS 23, MS 24, MS 26, MS 27, MS 28, MS 30, MS 31, MS 33, MS 34, and MS 36. Comparison was made with previously obtained profiles of cattle, sheep, and goat strains performed at WBVR using MLVA.

##### 2.1.4.2 Whole-genome sequencing and annotation

Sample M25157 preparation of the D3 abortion case involved a 10% placenta tissue suspension that was filtered and differentially centrifuged. DNA extraction was done with the Qiagen blood & tissue kit following the manufacturer’s instructions. Library prep was done with Oxford Nanopore Technologies Ligation Sequencing Kit V14 (SQK-LSK114), and long read sequencing was performed using PRO10.4.1 flow cell on a Nanopore PromethION 24 (Nanopore Technologies, Oxford). Draft genome assembly was performed by a focused approach. A custom *C. burnetii* database was constructed from *C. burnetii* sequences gathered from NCBI from all available whole genomes (13 June 2025). Raw reads were aligned and selected using the custom *C. burnetii* database. Genome assembly and polishing were performed using (17). Assemblies were reoriented with dnaapler (v1.3.0.) (18) and annotated with Bakta (v1.11.4; database v6.0, full) (19). The number of IS1111A elements per genome was determined by a tblastn (Protein Query-Translated Subject BLAST 2.17.0+) (20). The first IS1111A copy from the *C. burnetii* RSA 493 genome was used as the reference (NCBI accession: NP_819095.3), only hits with at least a 95% length coverage were counted.

##### 2.1.4.3 Genomic comparisons

A core genome multi-locus sequence typing (cgMLST) cluster analysis was performed within Ridom Seqsphere+ (v10.5.4) (21) with our data and data from *C. burnetii* isolates from the NCBI database; details of isolates in selection can be found in the Supplementary Table S2. Isolates were selected based on genome completeness and isolation associated with clinical disease in a variety of species: cattle, goat, sheep, and human. Within Ridom Seqsphere+ a new template was created with reference genome *C. burnetii* RSA 493/Nine Mile Phase I (NCBI accession: NC_002971). All gene features were selected, default checks were done, and all genes creating warnings were removed. This resulted in a core genome of 1,302 genes on which the cgMLST was based. Full genome alignments were made with progressiveMauve (build date 25 Feb 2026 at 13:36:49) (22) and displayed using pyGenomeViz (v1.6.1) (23).

### 2.2 Humans

#### 2.2.1 Risk assessment of human exposure

In line with the Dutch Response Structure for Zoonoses a coordination meeting was organized by the National Institute for Public Health and the Environment (RIVM) to perform a risk analysis for human exposure (24). As a result of the risk assessment, visitors, residents, and employees were considered as groups potentially exposed to *C. burnetii*. Risk assessment was based on the degree of direct contact with potentially infected material such as placentas, birth fluids, or aborted fetuses. Employees were classified as high-risk contacts, the visitors as low-risk contacts, and the residents of the surrounding area in Limburg as negligible-risk contacts. All high-risk contacts (*n* = 60) were informed and invited for Q fever serological screening and a questionnaire. Questionnaire data on level of involvement with the animals, type and onset of symptoms were collected among the group of employees who were tested. Additional questions were asked about the type of work at the zoo, history of Q fever, potential symptoms and risk factors for developing severe or chronic disease from Q fever.

Additionally, zoo visitors were notified through media communications and advised to contact their general practitioner if they developed any symptoms of acute Q fever following their visit to the zoo. General practitioners in the region were informed and advised to test patients that would report themselves with symptoms after a visit to the zoo or when living in close proximity. Authorities in the neighboring German and Belgian regions were informed because of the cross-border visitor population, so they could ensure that potentially exposed individuals from those countries were also informed.

#### 2.2.2 Human case definition

Human cases of Q fever were defined according to the national guidelines (25). A confirmed case was defined as an individual who had visited the zoo or lived in close proximity between 29 April and 1 June and showed clinical symptoms compatible with Q fever (fever, pneumonia, and/or hepatitis) combined with a serologically confirmed infection or detected *C. burnetii* DNA in a blood sample via PCR.

A probable case was defined as an individual who had visited the zoo in the relevant exposure period and showed clinical symptoms compatible with Q fever combined with a single positive serological test without yet documented seroconversion or PCR confirmation. A possible case was defined as an individual who had visited the zoo in the relevant exposure period and showed clinical symptoms compatible with Q fever but lacked laboratory confirmation or serological evidence.

#### 2.2.3 Diagnostic testing of humans

Caretakers and veterinarians (*n* = 34) of the zoo were tested by the GGD South Limburg for *C. burnetii* serology, with 3- to 4-week intervals. Serological samples were analyzed for phase I and phase II IgG and phase I and phase II IgM by immunofluorescence assay (IFA) on initial testing. Testing was repeated (phase I and II IgG and phase II IgM IFA) within 3- to 4-week intervals to detect seroconversion. Patient serum samples of 500 microliters were analyzed in duplicate. Samples were defined as ‘positive’ by cut-off titer ≥1:32. Laboratory tests were performed according to manufacturers’ protocols (Focus Diagnostics, USA).

PCR was used to test people who were symptomatic at the moment of testing to exclude pre-seroconversion infection in seronegative subjects. PCR was targeted at the gene IS1111. A Cycle threshold (Ct) value of <45.0 was considered positive (26). Individuals who were already tested by PCR for active infection were excluded from subsequent rounds of testing, just like individuals with a documented history of Q fever. Acute infections were defined by IgG seroconversion, or presence of anti-Coxiella phase II IgM titer, or presence of *C. burnetii* in serum tested by PCR.

## 3 Results

### 3.1 Animals

#### 3.1.1 Case handling in zoo animals

##### 3.1.1.1 Finnish forest reindeer

During the calving season of a group of six pregnant Finnish forest reindeer, four animals experienced abortion. Clinical symptoms were associated with dystocia and non-progressing parturition; details are described in Supplementary Table S1. One animal (D5) gave birth to a weak calf with neonatal joint hyperextension secondary to flexor tendon laxity. The calf was able to drink and to follow the mother and the herd. Subsequent development of this calf was normal. None of the does that aborted had systemic illness. Mild vaginal discharge was noted post-abortion. During the first field visit, all does were officially sampled for shedding, vaccinated with Coxevac®, and treated with long-acting oxytetracycline as a preventive herd-level measure. The reindeer had not received previous vaccination against *C. burnetii*. The last pregnant doe (D6) was euthanized as a precautionary measure to prevent possible shedding of *C. burnetii* after parturition.

##### 3.1.1.2 Other susceptible animals in the zoo

An inventory was made of animal species known to be susceptible in the zoo, such as the camelids, vicuñas, goats, giraffes, kudus, gazelles, and Highland cattle. Extra attention was paid to clinical signs in susceptible animal species. As a precautionary measure, all ruminants were vaccinated with Coxevac® after the field visit. Goats that were present in the zoo were previously vaccinated against *C. burnetii* according to the mandatory vaccination program in the Netherlands. Other animals present in the zoo were not vaccinated against *C. burnetii.* A review of historical data to identify any previous related incidents and a retrospective analysis of the Finnish forest reindeer populations to determine evidence of prior infection or circulation of the pathogen did not provide indications of prior *C. burnetii* infection.

#### 3.1.2 Laboratory results for animals

Diagnostic sampling had positive antigen detection with qPCR from vaginal swabs and positive serology (ELISA) in all cases of abortion (D1–D4) and in one animal (D5) after normal parturition. Placental tissue and fetal liver tissue (D3) retrieved after abortion were positive for *C. burnetii* infection by qPCR. Following necropsy of a young female (D7), the vaginal swab and tissue samples from the spleen and liver resulted in positive antigen detection with qPCR and negative qPCR from uterus tissue. Isolation and whole-genome sequencing from diagnostic placental material D3 was successful. Historical serum samples from the herd of reindeer (including two animals that had moved to another zoo) were all (*n* = 13) negative for antibody analysis (ELISA), see Supplementary Table S1.

#### 3.1.3 Pathological examination

Pathological examination was performed on the fetus and placenta from D3 at VPDC; no macroscopic lesions were identified. In histopathology, mild interstitial edema was seen with an occasional focal neutrophil. Immunohistology revealed focal granular intracytoplasmic staining for *C. burnetii* antigen in occasional trophoblasts in the placenta (Supplementary Presentation S1). Necropsy of a young doe (D7) was performed at WBVR. Necropsy revealed no macroscopic lesions. Organ tissue samples from the spleen, uterus, liver, and vaginal swab were collected for antigen detection by qPCR analysis.

#### 3.1.4 Molecular identification

##### 3.1.4.1 Multilocus variable-number tandem repeat analysis (MLVA)

MLVA results of samples D1–D5 (Supplementary Table S3) show an incomplete profile of PCR products. D1, D2, and D4 show an identical number of repeats at 7 out of 14 MS with a cattle strain (cattle GB) and show a different profile compared with the outbreak goat strain (CbNl01).

##### 3.1.4.2 Whole genome sequencing (WGS)

The Finnish forest reindeer strain (M25157) from placenta D3 was isolated and sequenced. Together with the goat strain (3262) and the cattle strain (2574) from the Netherlands, they were annotated, and the number of IS1111 elements was determined (Table 1). The annotations of the chromosome of M25157 and the Dutch cattle strain (2574) had high similarity in all genome characteristics. However, the Dutch goat strain (3262) differed from M25157 as it is larger and has a higher number of genes, more coding DNA sequences, more pseudogenes, and a higher number of IS1111 elements. This difference between the three isolates was not seen in the plasmids (see Table 1).

**Table 1:** Genome and annotation characteristics of chromosomes and plasmids of *Coxiella burnetii* isolates.

Characteristics	Chromosome			Plasmid		
	M25157	3262	2574	M25157	3262	2574
	Reindeer	Goat	Cow	Reindeer	Goat	Cow
Sequence(s)						
Length	1,993,772	2,081,202	1,993,827	37,327	37,320	37,330
Count	1	1	1	1	1	1
GC	42.7	42.9	42.7	39.3	39.3	39.3
N50	1,993,772	2,081,202	1,993,827	37,327	37,320	37,330
N90	1,993,772	2,081,202	1,993,827	37,327	37,320	37,330
N ratio	0	0	0	0	0	0
Coding density	89.3	89.7	89.4	81.3	80.6	81.3
Annotation						
tRNAs	43	43	43	0	0	0
tmRNAs	1	1	1	0	0	0
rRNAs	3	3	3	0	0	0
ncRNAs	17	17	17	0	0	0
ncRNA regions	2	2	2	0	0	0
CRISPR arrays	0	0	0	0	0	0
CDSs	2027	2191	2025	48	49	48
Pseudogenes	83	123	82	2	2	2
Hypotheticals	317	411	319	19	24	19
sORFs	0	0	0	0	0	0
Gaps	0	0	0	0	0	0
oriCs	0	0	0	0	0	0
oriVs	0	0	0	0	0	0
oriTs	0	0	0	0	0	0
IS1111	21	86	21	0	0	0

##### 3.1.4.3 Genomic comparisons

**Figure 2:**
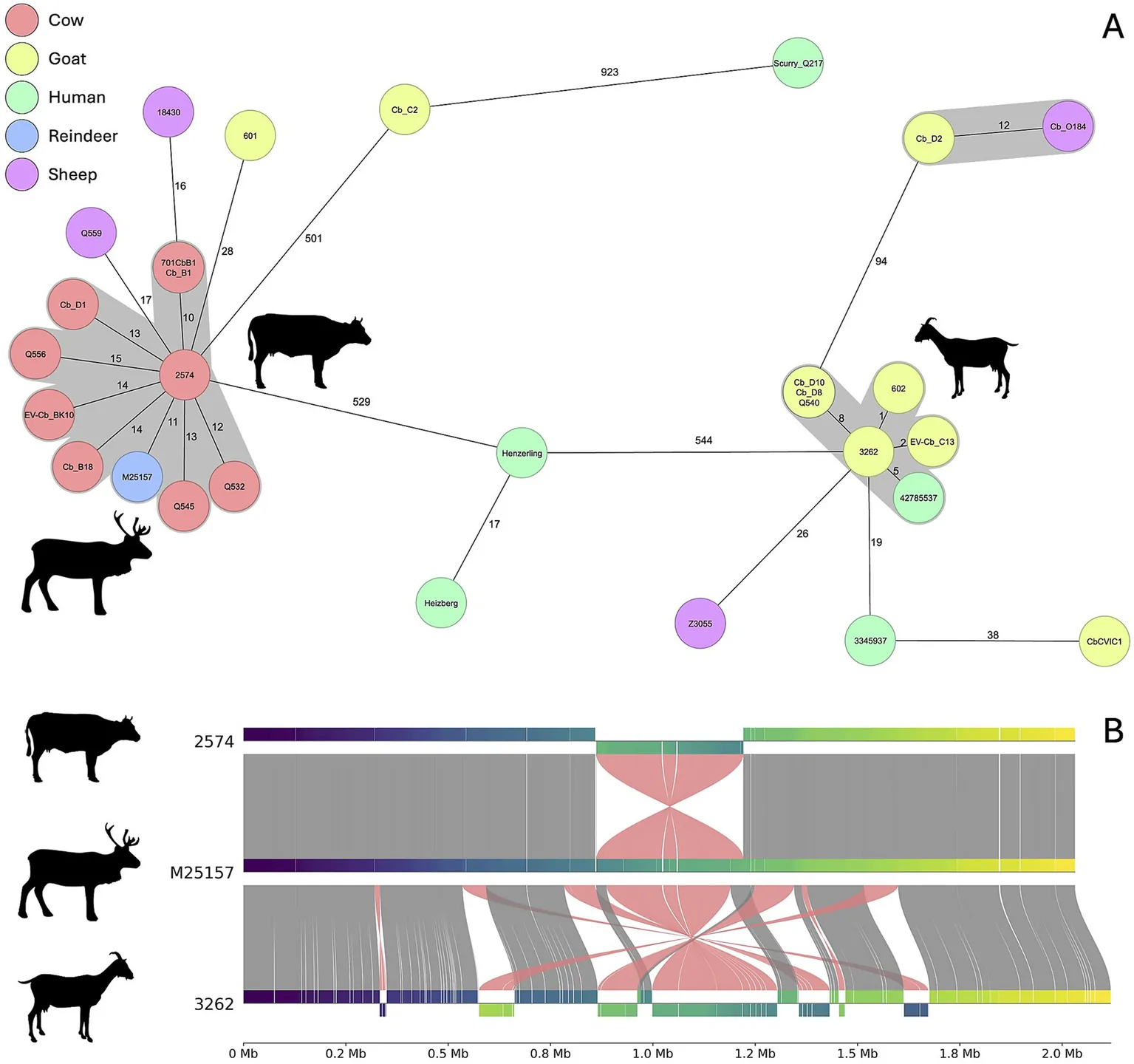
Genomic comparisons of *Coxiella burnetii* strains. **(A)** CgMLST cluster analysis of 29 *C. burnetii* strains based on 1,302 loci. Isolates are colored by host species; numbers above branches indicate SNP differences. Gray clusters were defined using a 15-SNP threshold. **(B)** ProgressiveMauve alignment of three *C. burnetii* genomes showing locally collinear blocks (LCBs). Each horizontal panel represents one genome; colored LCBs denote homologous regions (height reflects sequence conservation). Blocks above the centerline indicate forward orientation relative to the reference; those below show reverse complement orientation. Inversions are highlighted in coral.

Core genome MLST (cgMLST) was performed on *C. burnetii* isolates from humans (*n* = 5), cattle (*n* = 8), goats (*n* = 8), sheep (*n* = 4), and reindeer (*n* = 1). The cgMLST cluster analysis visualized the reindeer isolate clusters with historical cattle strains. A human isolates cluster with goat isolates, and a human and sheep isolate show some similarity, see Figure 2A. The alignment of local collinear blocks reflected the same differences as seen for the genomic characteristics: only one inversion between the reindeer strain and the cattle strain, but a multitude of inversions between the reindeer and the goat strain (Figure 2B). Moreover, there were many small insertions in the goat strain compared to the other two strains.

### 3.2 Results: humans (questionnaire and serology)

Employees who were tested filled in the questionnaire on their first visit (*n* = 34); these consisted of 18 men and 16 women, see Table 2. Most employees (*n* = 20) indicated they had been in contact with the forest reindeer abortions or with birth products. Eight others indicated involvement in related activities such as aftercare or cleaning up the birth products. At the time of testing, 12 employees reported experiencing symptoms ranging from fatigue to headaches. Two of them described having a fever. Hepatitis and pneumonia were not reported.

**Table 2:** Results from the active case finding among employees (questionnaire and serology).

Results	*n* = 34
Male	18 (53%)
Female	16 (47%)
Age range	20–54 years
Contact with births, abortions, or related materials	28 (82%)
Symptoms reported	20 (59%)
History of Q fever infection	1 (3%)
Possible cases	6 (18%)
Probable cases	0 (0%)
Confirmed cases	0 (0%)

Of the 34 people tested, none met the case definition of a probable or confirmed case, as the total number of laboratory-confirmed acute Q fever infections was zero. Six of them met the criteria of a possible case, but all tested *C. burnetii* PCR negative. One of the employees reported a prior Q fever infection. Positive cases were required to be reported to the local GGD according to the national notification guidelines. However, no notifications were received during the aforementioned period. Furthermore, no related cases were reported from other regions of the Netherlands that could be epidemiologically linked to this zoo outbreak.

## 4 Discussion

In this case, abortion due to *C. burnetii* was confirmed in four Finnish forest reindeer in a zoo in the Netherlands. In response to this, a comprehensive package of control and mitigation measures was implemented. These measures included strict hygiene protocols, restriction of public access, and the use of personal protective equipment. For the animals, these measures were directed towards isolation of the animals, antibiotic therapy, manure destruction, vaccination, and precautionary euthanasia of one pregnant animal. Similar measures have been described during the 2007–2010 Q fever outbreak and are considered effective in limiting both animal-to-animal and animal-to-human transmission (1, 27, 28). Vaccination was applied in a variety of ruminants in the zoo, although there were concerns regarding effectiveness and safety, as the vaccine (Coxevac®, CEVA Sante Animale) is only registered for use in cattle, goats, and sheep. Although the effectiveness of antibiotic treatment in reducing shedding is controversial, from a precautionary point of view, all female reindeer that aborted or had a parturition were treated with long-acting oxytetracycline (10).

Despite shedding of *C. burnetii* in the Finnish forest reindeer, likely subsequent environmental contamination, and, thus, human exposure, no human cases were identified, including the persons who were directly in contact with the reindeer and with birth products. Several factors may explain the absence of human cases, including the immediate implementation of hygiene measures, consistent use of personal protective equipment, separation of the animals from the public, immediate removal and destruction of birth products, and disinfection of the location where birth materials were found. Other contributing factors may include a lower effective bacterial load, reduced environmental exposure, or the involvement of a strain with limited zoonotic potential. These observations are consistent with previous reports, which indicate that although the prevalence of *C. burnetii* in cattle is in many studies considerably higher than in goats and sheep, infected cattle are much less described as a source related to human outbreaks (29). Most human outbreaks are related to shedding sheep and goats (11). As it was unknown to which *C. burnetii* strains the Finnish forest reindeer infections would belong, it was important, due to the public function of the zoo, to carefully consider which control measures to implement.

The strain of *C. burnetii* causing abortions in Finnish forest reindeer was successfully identified using targeted diagnostic approaches. Shedding of the pathogen was demonstrated in all reindeer examined. The detection of *C. burnetii* in vaginal swabs and fetal and placental material, positive staining of placental tissue with immunohistology, and abortions confirms infection within the Finnish forest reindeer herd. *C. burnetii* has been demonstrated in many animal species but has not previously been reported in Finnish forest reindeer together with the associated abortions. One of the considerations in the risk-based approach was to investigate whether the *C. burnetii* strain found in the reindeer was similar to the goat strain previously linked to the Dutch Q fever outbreak. The first analysis with MLVA results showed a comparable profile with a historical cattle strain. WGS data of the full genome and plasmid were analyzed with cgMLST, and this indicated a close association with historical cattle strains. Annotation of the full genome and plasmid in this study demonstrated a high degree of similarity to the Finnish forest reindeer isolate and the historical cattle strain as well. The difference in genomic composition and multitude of inversions of the goat strain (3262) compared with the reindeer strain could be explained by the presence of additional genes in the goat strain, as described previously by Kuley et al. (13, 30); this might contribute to the virulence. These results together show that the *C. burnetii* strain found in the Finnish forest reindeer is different from the goat strain (3262) that was associated with the human Q fever outbreak in the Netherlands.

*Coxiella burnetii* is an almost ubiquitous pathogen that has been identified in a wide range of animal species in the Netherlands. Domestic ruminants, particularly sheep, goats, and cattle, are recognized as the primary reservoirs, and vaccination is mandatory for specific high-risk groups. However, the pathogen has also been detected in other animal species, including wildlife and ticks, indicating a broader ecological distribution. Prevalence of *C. burnetii* in wildlife or in ticks is not fully known. In Dutch wildlife, *C. burnetii* has been detected in roe deer and rats (31, 32), and in ticks, the prevalence was low: 0.2% (33). During the first field visit, Q fever experts concluded that source tracing for the infection would be very complex and expensive, while the chance of finding the source was very low since *C. burnetii* is a ubiquitous bacterium. Nevertheless, advice regarding additional diagnostics was provided in case of reproductive disorders in other animal species. Historical serological analyses of the herd of Finnish forest reindeer in the zoo showed no antibodies against *C. burnetii*. However, interpreting these historical serological results is challenging as antibody responses can be low or transient, and the timing of sampling relative to infection may further complicate assessment (3, 34).

This case highlights the challenges of decision-making in novel *C. burnetii* shedding and the need to balance control measures with the precautionary principle. Preventive measures were primarily designed for a virulent strain. *C. burnetii* strains are likely to circulate in domestic ruminants as well as in wildlife in the Netherlands. The relationship between genomic composition and virulence of different strains remains insufficiently determined, underscoring the need for further focused research in this area. Improved characterization of genetic determinants and molecular markers associated with virulence and zoonotic potential could, in the future, enable rapid and reliable screening of strains. Such advancements would facilitate the differentiation of strains, thereby informing risk assessment and enabling the implementation of targeted and proportionate control measures.

An effective response requires diagnostics, strain typing, and collaboration among One Health stakeholders. In case of abortion in animals, appropriate, accessible hygiene measures should be implemented immediately, including the proper removal and disposal of placentas. If shedding is detected, adequate human healthcare, with diagnostic testing and attention to Q fever, is essential to identify patients. Lessons from this case can guide future responses, helping to navigate uncertainty while protecting both animals and public health.

## Data Availability

The dataset on *C. burnetii* M25157 generated for this study has been deposited in the European Nucleotide Archive (ENA) at EMBL-EBI under accession number PRJEB112871.

## Supplementary material

Supplementary Table S1 Animal details and diagnostic results.

Supplementary Table S2 *Coxiella burnetii* genomes collected from GenBank for the cgMLST.

Supplementary Table S3 MLVA results.

Supplementary Presentation S4 Picture IHC positive staining in placental tissue.
